# Gynecomastia: Etiological Analysis Beyond Hormonal Imbalance

**DOI:** 10.3390/life16061010

**Published:** 2026-06-16

**Authors:** Jae Heon Kim, Miho Song, Kisoo Lee, Min Hyuk Lee, Yun Seob Song

**Affiliations:** 1 Department of Urology, Soonchunhyang University School of Medicine, Seoul 04401, Republic of Korea; piacekjh@hanmail.net (J.H.K.); miho@schmc.ac.kr (M.S.); 2 Department of Urology, Dong-A University College of Medicine, Busan 49201, Republic of Korea; kiddung@hanmail.net; 3 Department of Surgery, Soonchunhyang University School of Medicine, Seoul 04401, Republic of Korea

**Keywords:** gynecomastia, hormones, estradiol, testosterone, comorbidity, etiology, age, medications

## Abstract

**Background:** Gynecomastia is a benign enlargement of the male breast with multiple causes. This retrospective study evaluated whether serum estradiol (E2), testosterone (T), or the E2/T ratio differed between patients and controls, while explicitly accounting for the limited hormonal subset and lack of statistical significance. **Methods:** We retrospectively reviewed 68 men with benign gynecomastia (1996–2000) and 10 healthy controls. Analyses were conducted in two predefined populations: a full etiologic cohort (*n* = 68) and a hormonal-analysis subset (37 patients and 10 controls). Serum E2 and T were measured in 37 patients and all controls, and the E2/T ratio was calculated. The E2/T ratio was analyzed primarily as a continuous variable rather than relying solely on a control-derived threshold. **Results:** Gynecomastia was most frequent in adult and middle-aged men. Idiopathic cases were most common, followed by drug-related, hormonal, and disease-related causes. The mean E2/T ratio was higher in patients (12.0 ± 1.8) than controls (7.1 ± 0.5), but this difference did not reach statistical significance (E2: *p* = 0.21; T: *p* = 0.34; E2/T: *p* = 0.07). Among tested patients, 13.5% had low T, 24.3% high E2, and 43.2% elevated E2/T ratios. Liver disease and drugs such as H_2_ blockers and psychoactive agents contributed in some cases, while 50% were idiopathic. **Conclusions:** Because hormone comparisons were non-significant and based on a limited subset, these data do not support an independent association between gynecomastia and circulating E2, T, or the E2/T ratio. Clinical evaluation should consider age, comorbidities, and medications in addition to hormonal factors.

## 1. Introduction

Gynecomastia is a benign proliferation of male breast glandular tissue and is one of the most common breast conditions encountered in males [1,2]. It typically presents as unilateral or bilateral enlargement of palpable subareolar glandular tissue and may be transient or persistent [2]. Reported prevalence varies widely according to age, study population, and diagnostic criteria, ranging from subtle enlargement detectable only on physical examination to more prominent breast development approaching a female-like contour [1,2,3,4,5]. Although often physiological and self-limited, gynecomastia is clinically important because it may produce tenderness, cosmetic concern, and psychological distress, and it may occasionally reflect an underlying endocrine, systemic, neoplastic, or pharmacologic disorder [2,3,4,5,6,7,8].

The pathophysiology of gynecomastia is classically attributed to an imbalance between estrogenic stimulation and androgenic inhibition at the level of breast tissue. Increased circulating estrogen, enhanced peripheral aromatization of androgens, reduced androgen production, or impaired androgen action can shift this balance toward ductal epithelial and stromal proliferation [7,8,9]. Previous clinical studies have described elevated estradiol levels, lower testosterone levels, and increased estradiol/testosterone ratios in subsets of affected patients [9]. However, these biochemical abnormalities are not uniformly observed, suggesting that systemic hormone concentrations alone may not fully explain the disorder.

Etiology also varies substantially by age group. Prepubertal gynecomastia is uncommon and should prompt careful evaluation because it may be associated with endocrine abnormalities or neoplastic conditions [10,11]. In contrast, pubertal gynecomastia is common and usually transient, reflecting temporary hormonal disequilibrium during adolescence [4,12,13,14]. In adult men, the differential diagnosis broadens to include testicular tumors, chronic systemic disease, medication effects, and age-related endocrine change [15,16]. Familial clustering and otherwise unexplained cases have also been reported, implying that genetic susceptibility or altered tissue responsiveness may contribute in selected individuals [17,18,19].

More recent clinical reviews have emphasized that gynecomastia should not be regarded solely as a disorder of measurable serum hormone imbalance. Contemporary evidence indicates that the presence and severity of gynecomastia do not consistently correlate with circulating estradiol or testosterone concentrations, highlighting the importance of clinical context, tissue-level responsiveness, and non-hormonal contributors [20,21,22,23,24]. Drug-induced gynecomastia has received particular attention, with strong or probable associations reported for antiandrogens, spironolactone, cimetidine, 5-alpha reductase inhibitors, and several prolactin-elevating agents [25,26,27,28]. In addition, broader endocrine and epidemiologic studies continue to support the roles of age-related endocrine change, medication burden, and heterogeneous adult etiologic patterns [27,28,29,30].

Given these considerations, we investigated the relative roles of hormone profiles, medication use, and comorbidities in patients with benign gynecomastia. Specifically, we assessed whether serum estradiol, testosterone, or the E2/T ratio differed between patients and controls, and we examined how major etiologic categories varied according to age. Rather than re-establishing hormonal theory, this study aims to clarify how age, medication exposure, comorbidities, and limited hormonal data intersect within a retrospective clinical cohort.

## 2. Materials and Methods

### 2.1. Study Population

We retrospectively reviewed medical records of male patients who presented with breast enlargement and were diagnosed with gynecomastia at our institution between December 1996 and August 2000. Diagnostic evaluation included mammography, breast ultrasonography, and fine-needle aspiration or excisional biopsy. Only cases confirmed as benign gynecomastia on cytology or histology—defined as glandular proliferation without evidence of malignancy—were included. A total of 68 patients met the inclusion criteria. For comparison, 10 adult men without gynecomastia, who visited the hospital for unrelated minor conditions during the same period, served as controls.

The study was approved by the Institutional Review Board of Soonchunhyang University Seoul Hospital (Reg. No. 2025-12-009).

### 2.2. Data Collection

Clinical information was obtained through patient interviews and chart review, including age, medical history, medication use, systemic diseases, and family history of gynecomastia. All patients underwent complete physical examination. Hormone assays were performed in a subset of participants; serum estradiol (E2) and testosterone (T) levels were available for 37 of the 68 gynecomastia patients and for all 10 controls. E2 was measured in pg/mL and T in ng/mL. Blood-sampling time for hormone assays was not consistently retrievable from retrospective records and was therefore not standardized.

### 2.3. Definition of Hormone Reference Ranges

Reference ranges for adult male serum estradiol (15–60 pg/mL) and testosterone (2.7–10.7 ng/mL) were based on standard laboratory values. Because no universally accepted biologic reference interval exists for the estradiol/testosterone ratio (E2/T), the control-derived range was treated as exploratory. In the revised analysis, the E2/T ratio was analyzed primarily as a continuous variable, and any threshold-based categorization was used only for descriptive purposes and not for inferential conclusions. To normalize units and improve interpretability, the E2/T ratio was calculated as E2 (pg/mL) ÷ T (ng/mL) × 1000. Therefore, the reported E2/T values represent scaled ratios rather than unitless raw quotients. From the control mean ± 1.96·SD, the 95% confidence interval yielded a reference range of 6.1–8.1. An E2/T ratio > 8.1 was considered elevated, while <6.1 was considered low.

### 2.4. Evaluation for Underlying Causes

All patients were systematically evaluated for potential etiologies. Abdominal ultrasonography was performed to detect adrenal abnormalities, including feminizing adrenal tumors. Careful testicular examinations were conducted, with ultrasonography performed when clinically indicated to exclude tumors or severe atrophy. Thyroid function and liver function tests were also obtained to identify hyperthyroidism or hepatic dysfunction as possible contributors.

Each case was categorized into one of four etiological groups:

• Hormonal: primary cause attributed to abnormal sex hormone profile (e.g., elevated E2/T ratio or endocrine disorder).
• Drug-related: attributed to medications known to induce gynecomastia.
• Disease-related: associated with systemic or organ disease (e.g., liver disease, renal failure, or endocrine disorders).
• Idiopathic: no identifiable cause, with normal hormone levels and no relevant medications or diseases.

When multiple potential causes were present, a hierarchical classification was applied. Cases with abnormal hormone profiles were categorized as hormonal, even if medications or comorbidities were also present. This ensured each patient was assigned to a single etiological group.

### 2.5. Statistical Analysis

Continuous variables (e.g., hormone levels, age) are expressed as mean ± standard error of the mean (SEM). Hormone levels between gynecomastia patients and controls were compared using the non-parametric Mann–Whitney U test due to small sample size and non-normal data distribution. A *p*-value < 0.05 was considered statistically significant. Statistical analyses were conducted using standard software.

### 2.6. Analytic Populations

To improve interpretability, analyses were conducted in two predefined populations: (1) the full clinical cohort (*n* = 68), used for descriptive analyses of age, medications, comorbidities, and etiologic distribution, and (2) the hormonal-analysis subset (37 gynecomastia patients and 10 controls), used for E2, T, and E2/T comparisons. To assess potential selection bias, patients with available hormonal data (*n* = 37) were compared with those without (*n* = 31) across key characteristics. The mean age was 43.8 ± 3.1 years in the tested group and 44.9 ± 3.8 years in the untested group (*p* = 0.82). The proportions with medication exposure were 32.4% vs. 25.8% (*p* = 0.54), and those with comorbidities were 29.7% vs. 25.8% (*p* = 0.71). Etiologic distribution was also similar between groups (*p* = 0.68 by Fisher’s exact test). Although no statistically significant differences were observed, this assessment is limited by sample size and the comparison should be interpreted cautiously.

### 2.7. Overlapping Etiologies

The original mutually exclusive etiologic classification was retained for comparability with the historical dataset. In a secondary descriptive assessment, contributors were also examined in a non-mutually exclusive manner, indicating that medication exposure, comorbid disease, and hormonal abnormalities frequently co-occurred within the same patient; however, these findings are presented descriptively due to limited sample size.

## 3. Results

### 3.1. Patient Demographics

We analyzed 68 men with gynecomastia and 10 male controls. The mean age of patients was 44.3 ± 2.5 years, comparable to controls (45.6 ± 5.4 years, *p* = 0.78).

### 3.2. Age Distribution

Gynecomastia occurred across all ages but was most common in adulthood. Only 1 case (1.5%) was prepubertal and 6 (8.8%) were adolescents. Most patients were adults: 30 (44.1%) were 18–49 years, 23 (33.8%) were 50–70 years, and 8 (11.8%) were ≥71 years (Table 1).

### 3.3. Causes by Age Group

Etiology varied with age (Table 1). The prepubertal case was hormonal. Among adolescents, 5 (83.3%) were idiopathic and 1 (16.7%) drug-related. In young adults (18–49 years), idiopathic causes predominated (66.7%), with smaller proportions hormonal (16.7%), drug-related (10.0%), and disease-related (6.7%). In middle-aged patients (50–70 years), drug-related causes were most frequent (43.5%), followed by idiopathic (26.1%), disease-related (17.4%), and hormonal (13.0%). In elderly patients (≥71 years), idiopathic and drug-related causes were equally common (37.5% each). Overall, idiopathic cases accounted for 50.0%, drug-related for 25.0%, hormonal for 14.7%, and disease-related for 10.3%. Formal statistical comparison across all five age groups was not feasible due to sparse cell counts in several strata (e.g., prepubertal *n* = 1, pubertal *n* = 6). To partially address this, a two-group comparison was performed collapsing younger patients (≤49 years, *n* = 37) versus older patients (≥50 years, *n* = 31). Drug-related causes were significantly more frequent in older patients (41.9% vs. 10.8%, Fisher’s exact test *p* = 0.005), while idiopathic causes were more frequent in younger patients (64.9% vs. 29.0%, *p* = 0.004). Because the full five-way age-group comparison with sparse cells remained statistically untenable, the detailed age-group patterns in Table 1 should continue to be interpreted as descriptive and exploratory rather than confirmatory.

### 3.4. Hormone Levels

**Figure 1:**
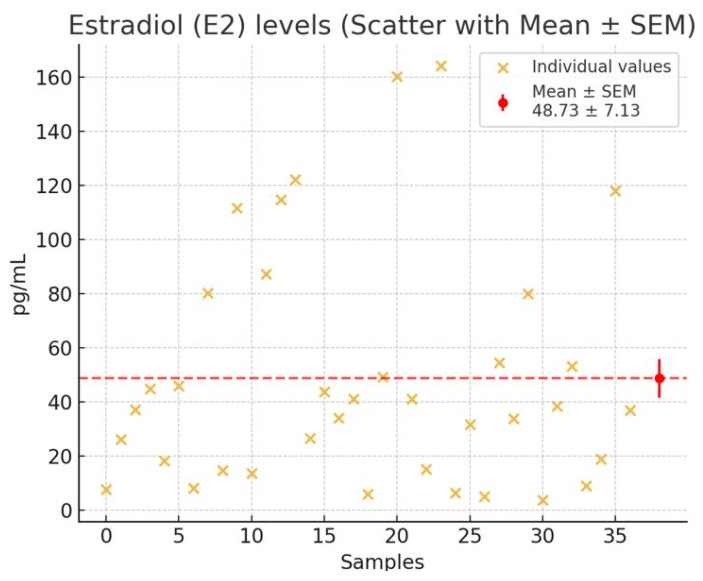
Scatter plot of serum estradiol (E2) levels in gynecomastia patients and controls. Individual values are shown with group means ± SEM. Patients had numerically higher E2 (48.7 ± 7.1 pg/mL) than controls (35.3 ± 3.9 pg/mL); this difference did not reach statistical significance (*p* = 0.21, Mann–Whitney U test).

**Figure 2:**
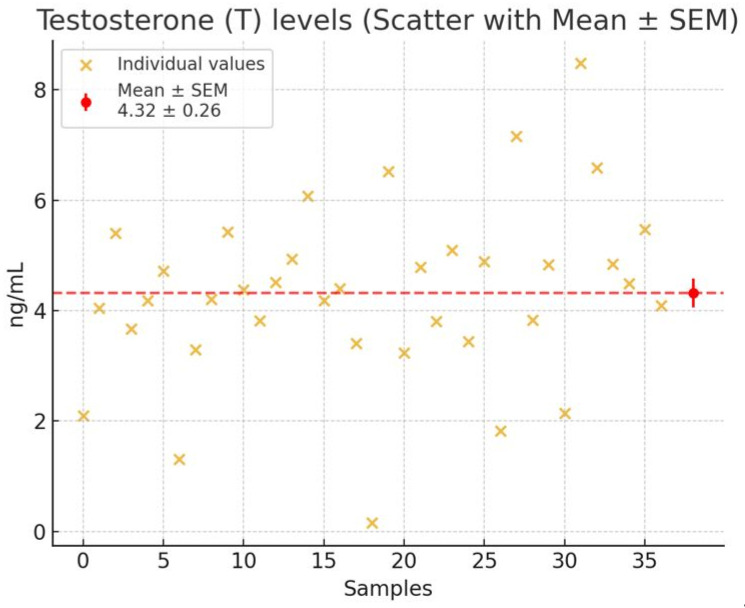
Scatter plot of serum testosterone (T) levels in gynecomastia patients and controls. Each dot represents an individual value with group means ± SEM. T was numerically lower in patients (4.3 ± 0.3 ng/mL) than controls (5.0 ± 0.4 ng/mL); this difference did not reach statistical significance (*p* = 0.34, Mann–Whitney U test).

**Figure 3:**
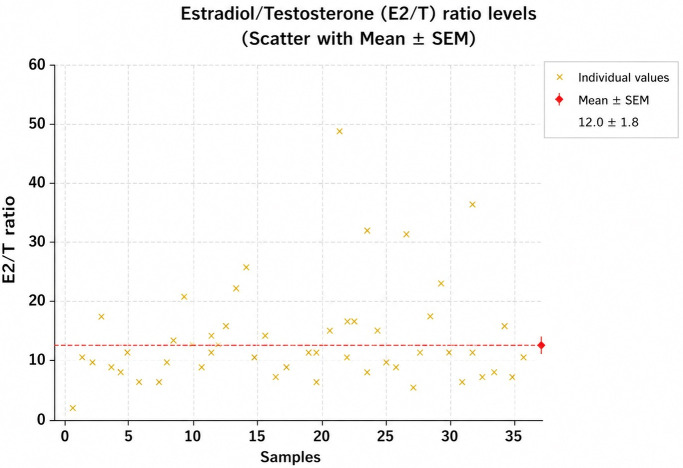
Scatter plot of the serum estradiol/testosterone (E2/T) ratio in gynecomastia patients and controls. Individual values are shown together with group means ± SEM. The E2/T ratio was numerically higher in patients (12.0 ± 1.8) than controls (7.1 ± 0.5); this difference did not reach statistical significance (*p* = 0.07, Mann–Whitney U test).

Hormone data were available for 37 patients and all 10 controls. Compared to controls, patients showed numerically higher estradiol (48.7 ± 7.1 vs. 35.3 ± 3.9 pg/mL, Figure 1), numerically lower testosterone (4.3 ± 0.3 vs. 5.0 ± 0.4 ng/mL, Figure 2), and numerically higher E2/T ratio (12.0 ± 1.8 vs. 7.1 ± 0.5, Figure 3), though none of these differences reached statistical significance (E2: *p* = 0.21; T: *p* = 0.34; E2/T ratio: *p* = 0.07; all by Mann–Whitney U test). Individual variability was marked: 13.5% had low testosterone, 24.3% had low estradiol, and 24.3% had high estradiol. Based on control-derived 95% CI, 43.2% had elevated E2/T ratios and 32.4% had low ratios (Table 2). No patients showed testosterone excess, testicular atrophy, or testicular tumors. Among the 37 patients who underwent hormonal testing, descriptive subgroup analyses were performed according to medication exposure and comorbidity status. Patients with medication exposure (*n* = 12) showed a numerically higher mean E2/T ratio compared with those without medication exposure (13.8 ± 2.4 vs. 11.2 ± 2.1), although this difference was not statistically significant (*p* = 0.41, Mann–Whitney U test). Similarly, patients with comorbidities (*n* = 11) demonstrated numerically higher serum estradiol levels than those without comorbidities (54.2 ± 12.1 pg/mL vs. 46.3 ± 8.3 pg/mL), but the difference did not reach statistical significance (*p* = 0.57). Because of the limited sample size, these subgroup findings should be interpreted as descriptive and hypothesis-generating rather than confirmatory.

### 3.5. Comorbidities

Nineteen patients (27.9%) had significant comorbidities, most commonly chronic liver disease (12 cases). Others included chronic respiratory disease (4), lung cancer (1), chronic renal failure (1), and prostate cancer on androgen-deprivation therapy (1). No cases of hyperthyroidism, Klinefelter syndrome, adrenal tumors, or other malignancies were identified.

### 3.6. Medication Use

Twenty patients (29.4%) were on drugs linked to gynecomastia. The most frequent were H_2_-receptor antagonists (12), followed by diazepam (6), theophylline (4), diuretics (1), perphenazine (1), tricyclic antidepressants (1), and anti-androgen therapy (1). Six patients (30%) were on multiple potential offending agents.

### 3.7. Idiopathic Cases

In 34 patients (50.0%), no cause was identified, making idiopathic gynecomastia the most common category despite thorough evaluation.

## 4. Discussion

Gynecomastia has long been conceptualized as a disorder resulting from an imbalance between estrogenic and androgenic activity at the level of breast tissue [7,8,9]. In adult males, the majority of circulating testosterone is produced by the testes, whereas estradiol is primarily generated through peripheral aromatization of testosterone and androstenedione in adipose and other tissues [9,16]. Under normal physiological conditions, a balance between these hormonal influences maintains minimal breast tissue proliferation. Disruption of this balance—whether through increased estrogen activity, reduced androgen action, or both—has traditionally been considered the central mechanism underlying gynecomastia.

However, the findings of the present study indicate that this classical paradigm alone may be insufficient to explain the clinical heterogeneity observed in gynecomastia. Although mean estradiol levels and E2/T ratios were numerically higher and testosterone levels were numerically lower in patients compared with controls, none of these differences reached statistical significance. These findings suggest that circulating hormone concentrations alone may not adequately account for the development of gynecomastia in this cohort.

Importantly, the absence of statistically significant differences should be interpreted with caution. Given that hormone measurements were available for only a subset of patients (37 of 68) and the control group was relatively small (*n* = 10), the study may be underpowered to detect modest but clinically relevant differences. Therefore, these results should not be interpreted as evidence against a hormonal contribution, but rather as indicating that systemic hormone levels alone are insufficient to fully explain the condition.

This interpretation is consistent with contemporary literature emphasizing that circulating hormone levels may not accurately reflect local tissue-level effects or receptor sensitivity [20,21,22,23]. In this context, it is increasingly recognized that local breast-tissue factors, including aromatase activity and receptor-mediated sensitivity, may play a critical role in modulating estrogenic effects. Intramammary conversion of androgens to estrogens and variation in androgen receptor responsiveness may lead to tissue-level hormonal imbalance even in the absence of measurable systemic abnormalities. Because these mechanisms were not directly assessed in this study, they should be considered hypothesis-generating rather than conclusive.

Age-related variation in etiology was a prominent finding in our cohort and further supports the multifactorial nature of the condition. Prepubertal gynecomastia was rare but clinically significant, as it may be associated with endocrine disorders or neoplastic conditions requiring prompt evaluation [2,10,11]. In contrast, pubertal gynecomastia was common and predominantly idiopathic, reflecting transient hormonal fluctuations during adolescence [2,4,12,13,14,26,29]. These findings align with established epidemiological data and reinforce the concept that physiological hormonal variability plays a key role in younger populations.

In adult patients, particularly those aged 18–49 years, idiopathic gynecomastia remained the most common category. Although serious underlying conditions such as testicular tumors or chromosomal abnormalities must be considered, none were identified in our study population [2,8,15]. In older individuals, drug-related and idiopathic causes predominated. Age-related endocrine changes—including declining testosterone levels, increased sex hormone–binding globulin, and enhanced aromatase activity—are known to shift the estrogen–androgen balance [16]. However, our findings indicate that these systemic changes alone do not consistently translate into measurable differences in circulating hormone levels, suggesting that additional factors such as tissue sensitivity and pharmacologic influences may be critical determinants.

However, in the present study, these age-related patterns were primarily descriptive, and formal statistical comparisons should be interpreted cautiously due to small sample sizes in several strata. Accordingly, these observations should be considered exploratory rather than confirmatory.

Medication exposure emerged as a major contributing factor in our cohort, accounting for approximately one-quarter of cases. H_2_-receptor antagonists, benzodiazepines, and methylxanthines were the most frequently implicated agents. These medications may induce gynecomastia through multiple mechanisms, including inhibition of androgen action, increased estrogen activity, or modulation of prolactin secretion [2,6]. Recent large-scale pharmacovigilance and systematic reviews have further highlighted the significant role of medications in the pathogenesis of gynecomastia, particularly in aging populations with polypharmacy [25,27,28]. These findings underscore the importance of detailed medication history in clinical evaluation.

Systemic diseases were identified in a subset of patients, with chronic liver disease being the most common. The liver plays a central role in steroid metabolism and hormone clearance; thus, hepatic dysfunction can result in altered estrogen–androgen balance [2]. Although less frequent, other conditions such as renal failure, malignancy, and chronic illness have also been associated with gynecomastia in previous studies [15,17,18,19]. In our cohort, these factors contributed to a minority of cases but remain clinically important considerations.

Notably, idiopathic gynecomastia accounted for approximately half of all cases, despite comprehensive evaluation. This finding is consistent with prior reports and highlights a critical limitation in current understanding of the condition [19,30]. Idiopathic cases may reflect subtle hormonal perturbations, increased local aromatase activity, altered receptor sensitivity, or genetic predisposition. These mechanisms are not captured by routine clinical testing, suggesting that gynecomastia may, in many cases, represent a disorder of tissue-level regulation rather than systemic endocrine imbalance. Importantly, because hormone testing was not performed in all patients (available for 37 of 68), some cases classified as idiopathic may in fact harbor underlying hormonal abnormalities that went undetected. This represents a meaningful limitation of the idiopathic categorization, and future prospective studies should ensure complete hormonal evaluation in all participants to minimize this source of misclassification.

Furthermore, the interpretation of etiologic classification should be approached with caution. Because a hierarchical classification system was applied, cases with abnormal hormone profiles were categorized as hormonal even when medications or comorbidities were also present. To address this limitation, we performed a secondary descriptive analysis allowing overlapping etiologies. Among the 68 patients, 11 (16.2%) had concurrent medication exposure and comorbid disease; 8 (11.8%) had both hormonal abnormalities and medication exposure; and 5 (7.4%) had all three contributors simultaneously present. Overall, at least two contributing factors coexisted in 24 patients (35.3%). This finding further supports the concept that gynecomastia is a multifactorial condition rather than a single-cause disorder.

The present study has several limitations. The relatively small sample size for hormonal analysis and the limited control group may have reduced statistical power. In addition, the retrospective design limited the availability of complete endocrine data, including LH, FSH, prolactin, and SHBG, which are recommended in contemporary evaluation guidelines. Blood-sampling time was not standardized and may have contributed to variability in testosterone measurements. In addition, although Figure 1, Figure 2 and Figure 3 include both patient and control data, the control-group data were not visually emphasized as a clearly distinct graphical series, which may have limited visual differentiation and reduced clarity in the between-group comparisons shown in Figure 1, Figure 2 and Figure 3. Furthermore, the use of a control-derived E2/T threshold should be considered exploratory because no universally accepted biological cut-off exists. Potential selection bias arising from incomplete hormone testing could not be fully excluded, although this was partially addressed through subgroup comparison. Despite these limitations, our study provides a clinically relevant perspective by integrating hormonal, pharmacological, and systemic factors in a real-world patient population.

In particular, by explicitly distinguishing between the full clinical cohort and the hormonal-analysis subset, this study improves interpretability and highlights the limitations of relying solely on biochemical markers in the evaluation of gynecomastia.

In conclusion, gynecomastia should be regarded as a multifactorial condition rather than a disorder solely attributable to hormonal imbalance. While estrogen–androgen disequilibrium remains an important conceptual framework, it does not fully account for the majority of clinical cases. Age, medication exposure, systemic disease, and idiopathic mechanisms all contribute to its development. These findings support a more comprehensive and individualized approach to evaluation, emphasizing the importance of clinical context over isolated laboratory measurements.

## 5. Conclusions

In conclusion, gynecomastia is a multifactorial condition that cannot be explained solely by estradiol, testosterone, or their ratio. Its etiology varies with age, with idiopathic forms predominating in younger patients and medication-related causes more frequent in older individuals. In this study, although E2 and E2/T ratios were numerically higher and testosterone was lower in patients, none of these differences reached statistical significance. Given the limited hormonal subset and small control group, these findings should be interpreted cautiously and do not support an independent association based on circulating hormone levels alone. Rather, the results suggest that systemic hormone measurements are insufficient to explain gynecomastia, and that clinical evaluation should incorporate medications, comorbidities, and patient context.

## Figures and Tables

**Table 1:** Distribution of causes of gynecomastia by age group (*n* = 68). Idiopathic cases were most frequent overall, while drug-related causes predominated in middle-aged and elderly patients.

Age Group (years)	Hormonal	Drug-Related	Disease-Related	Idiopathic	Total Cases
Prepubertal (≤10)	1 (100) *	0 (0)	0 (0)	0 (0)	1 (100)
Pubertal (11–17)	0 (0)	1 (16.7)	0 (0)	5 (83.3)	6 (100)
Young Adult (18–49)	5 (16.7)	3 (10.0)	2 (6.7)	20 (66.7)	30 (100)
Middle-aged (50–70)	3 (13.0)	10 (43.5)	4 (17.4)	6 (26.1)	23 (100)
Elderly (≥71)	1 (12.5)	3 (37.5)	1 (12.5)	3 (37.5)	8 (100)
All Ages	10 (14.7)	17 (25.0)	7 (10.3)	34 (50.0)	68 (100)

* Number of patients (%).

**Table 2:** Hormonal profile of gynecomastia patients relative to normal ranges (*n* = 37).

Parameter	<Normal	Normal Range	>Normal	Total
Estradiol (E2)	9 (24.3) *	19 (51.4)	9 (24.3)	37 (100)
Testosterone (T)	5 (13.5)	32 (86.5)	0 (0)	37 (100)
Estradiol/Testosterone (E2/T) ratio	12 (32.4)	9 (24.3)	16 (43.2)	37 (100)

* Number of patients (%). Normal ranges. E2: 15–60 pg/mL; T: 2.7–10.7 ng/mL. E2/T ratio (calculated as E2 [pg/mL] ÷ T [ng/mL] × 1000): 6.1–8.1.

## Data Availability

The original contributions presented in this study are included in the article. Further inquiries can be directed to the corresponding authors.

## Abbreviations

The following abbreviations are used in this manuscript:

E2	Estradiol
T	Testosterone
E2/T	Estradiol-to-testosterone ratio
SEM	Standard error of the mean
IRB	Institutional Review Board
H_2_ blocker	Histamine-2 receptor antagonist
FNAB	Fine-needle aspiration biopsy
